# Dendritic Degeneration of Human Auditory Nerve Fibers and Its Impact on the Spiking Pattern Under Regular Conditions and During Cochlear Implant Stimulation

**DOI:** 10.3389/fnins.2020.599868

**Published:** 2020-11-19

**Authors:** Amirreza Heshmat, Sogand Sajedi, Lejo Johnson Chacko, Natalie Fischer, Anneliese Schrott-Fischer, Frank Rattay

**Affiliations:** ^1^Faculty of Mathematics and Geoinformation, Institute for Analysis and Scientific Computing, Vienna University of Technology, Vienna, Austria; ^2^Laboratory for Inner Ear Biology, Department of Otorhinolaryngology, Medical University of Innsbruck, Innsbruck, Austria

**Keywords:** auditory nerve, degeneration, dendrite, cochlear implant, computational model, electrical stimulation, spike conductance, G-ratio

## Abstract

Due to limitations of human *in vivo* studies, detailed computational models enable understanding the neural signaling in the degenerated auditory system and cochlear implants (CIs). Four human cochleae were used to quantify hearing levels depending on dendritic changes in diameter and myelination thickness from type I of the auditory nerve fibers (ANFs). Type I neurons transmit the auditory information as spiking pattern from the inner hair cells (IHCs) to the cochlear nucleus. The impact of dendrite diameter and degree of myelination on neural signal transmission was simulated for (1) synaptic excitation via IHCs and (2) stimulation from CI electrodes. An accurate three-dimensional human cochlear geometry, along with 30 auditory pathways, mimicked the CI environment. The excitation properties of electrical potential distribution induced by two CI were analyzed. Main findings: (1) The unimodal distribution of control dendrite diameters becomes multimodal for hearing loss cases; a group of thin dendrites with diameters between 0.3 and 1 μm with a peak at 0.5 μm appeared. (2) Postsynaptic currents from IHCs excite such thin dendrites easier and earlier than under control conditions. However, this advantage is lost as their conduction velocity decreases proportionally with the diameter and causes increased spike latency and jitter in soma and axon. Firing probability reduces through the soma passage due to the low intracellular current flow in thin dendrites during spiking. (3) Compared with dendrite diameter, variations in myelin thickness have a small impact on spiking performance. (4) Contrary to synaptic excitation, CIs cause several spike initiation sites in dendrite, soma region, and axon; moreover, fiber excitability reduces with fiber diameter. In a few cases, where weak stimuli elicit spikes of a target neuron (TN) in the axon, dendrite diameter reduction has no effect. However, in many cases, a spike in a TN is first initiated in the dendrite, and consequently, dendrite degeneration demands an increase in threshold currents. (5) Threshold currents of a TN and co-stimulation of degenerated ANFs in other frequency regions depend on the electrode position, including its distance to the outer wall, the cochlear turn, and the three-dimensional pathway of the TN.

## Introduction

**FIGURE 1:**
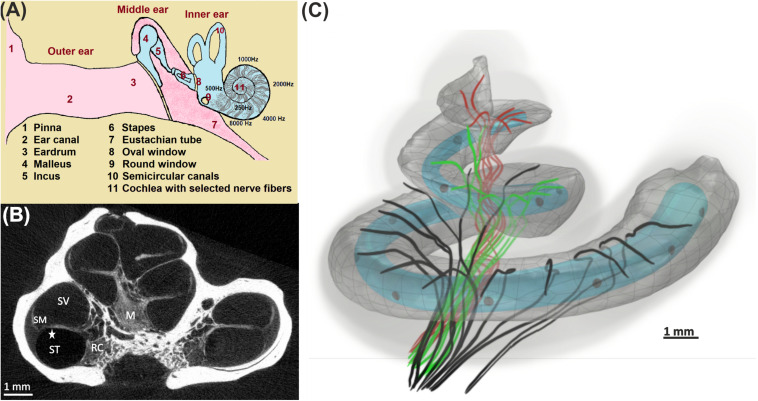
From acoustical to neural signal. **(A)** Ear anatomy; the acoustic signal enters the pinna, travels through the ear canal, and vibrates the eardrum. These vibrations are transferred by bony structures at the oval window to the fluid system in the cochlea, where they cause a local frequency mapping (tonotopical principle: each frequency has its place of resonance). **(B)** The fluid compartments scala tympani (ST), scala vestibuli (SV), and scala media (SM) coil up the bony modiolus (M). A spiral channel (Rosenthal’s canal, RC) within M resides somata of auditory nerve fibers that act as data lines between the hair cells in the organ of Corti (star) and higher processing centers in the brain. **(C)** 3D view of 30 differently colored fibers from the basal (black), middle (green), and apical (red) cochlea turns together with the scala tympani. An electrode array (gray circles) of a 12-channel cochlear implant (light blue; used for our simulations) is inserted in ST.

According to World Health Organization [WHO] (2020), nearly 466 million people worldwide have impairing hearing loss caused by hereditary, aging, disease, and injury. Hearing loss or deafness has several impacts on people’s daily life. One of the critical impacts of hearing loss is a lack of communication skills in the society. The ear is a vulnerable organ where sophisticated strategies were developed during evolution to transfer sound into neural signals (Figure 1) with high precision concerning frequency and loudness (Von Békésy and Wever, 1960; Mark and Rattay, 1990; Humes et al., 2010). In most cases, deficits in hearing performance are caused by a disturbed spiking pattern transmitted from the cochlea to the brain’s processing centers. Sensory hair cells in the cochlea convert sound into neural signals conducted along the auditory nerve fibers (ANFs) by two types of spiral ganglion neurons. The vast majority (about 95%) of spiral ganglion neurons are bipolar type I cells that connect inner hair cells (IHCs) via myelinated dendrites, large somata, and myelinated axons with the cochlear nuclei in the brain stem (Ota and Kimura, 1980). The myelination is essential for rapid action potential (AP) conduction along the ANF (Rattay et al., 2013). In contrast, smaller unmyelinated type II cells transmit APs from the outer hair cells (Spoendlin, 1985).

The somata of type I cells in humans are unmyelinated, whereas in non-primate mammals, they are densely myelinated (Thomsen, 1967; Spoendlin, 1972; Ota and Kimura, 1980; Arnold, 1987; Spoendlin and Schrott, 1989; Xing et al., 2012). Electrophysiological modeling studies demonstrate that reducing the number of membrane sheets covering the soma increases both the somatic membrane capacitance and conductance, which leads to a delay for the AP passing the soma (Rattay, 2000; Rattay et al., 2001). Depending on soma diameter and the number of sheets of covering glia cells, an AP needs about 200 μs longer (human vs. cat of regular type I cells) to pass the soma (Rattay et al., 2013). Particularly, the dendrite diameter plays an additional role when the neural signal has to cross the soma. The synaptic excitation might be easier for thinner fibers, yet their small inner-axonal current has to load the large soma capacitance. According to the simulations presented in section “Impact of DD and MT on Synaptic Threshold Current and AP Arrival Time in the Axon,” it is expected that a minimum dendrite diameter of about 0.5 μm is necessary for a safe AP conduction from the dendrite across the soma into the axon of a typical human type I cell.

Decoding the auditory information and its possible quality is based on the firing pattern delivered by the entity of type I cells (Miller and Sachs, 1983; Shamma, 1985; Rattay and Lutter, 1997). Their comprehensive innervation of all tonotopically organized cochlear regions is needed for perfect hearing. Moreover, small variations in type I fiber diameters with a common degree of myelination are ideal for precise spike arrival times in the next neural processing unit, the cochlear nucleus. Spoendlin published a narrow range of fiber diameters in children against a broader spectrum in adults. The group reported an enormous increase of percentage for the fibers smaller than 1 μm in hearing deficit specimens, suggesting that in terms of neural health, not only the number of fibers but also a normal range of diameters is one of the key elements for excellent hearing performance (Spoendlin and Schrott, 1989). In addition, several other components, such as stria vascularis, endocochlear potential, and hair cells, are essential for normal hearing (Keithley, 2020).

A cochlear implant (CI) is a medical device to help people with severe hearing impairment. Sophisticated strategies were developed to stimulate populations of ANFs externally via currents from an electrode array. One of the important parameters which influence the CI performance is auditory nerve condition. In a study by Blamey et al. (2012), CI recipients experienced a wide variety of performance, even with the same hearing loss levels. Shepherd and coworkers (Shepherd and Javel, 1997; Sly et al., 2007) reported a significant decrease in neural responses for degenerated ANFs compared with healthy cases suggesting that the condition of the auditory nerve could be a factor for extracellular stimulation outcomes. There is no reliable technique to estimate the outcomes of the CI in recipients before surgery because of many unknown factors that are involved. However, clinical studies showed that factors such as duration and age at onset of severe to profound hearing loss, age at implantation, etiology, using hearing aids, duration of moderate hearing loss, ANF degeneration, and residual hearing measures have some impact on CI performances (Gantz et al., 1993; Summerfield and Marshall, 1995; Waltzman et al., 1995; Albu and Babighian, 1997; Rubinstein et al., 1999; Friedland et al., 2003; Gomaa et al., 2003; Blamey et al., 2012; Lazard et al., 2012).

Although the degeneration pattern of IHCs and ANFs is not completely understood yet, it is related to the nature of the cochlear insult, the duration, and level of hearing loss. As an example, in a human cochlea with severe degeneration, Nadol reported more fibers in the proximity of Rosenthal’s canal than in the habenula perforate, suggesting the shortening of the dendrites in case of severe and profound degenerations. In addition, the number of axons was essentially higher than dendrites, and the number of somata was reduced to 10% of the normal range (Nadol, 1977). In case of age-related degenerations, some clinical studies of human temporal bones reported about 1000 to 1800 somata loss per decade that may contribute to reduction of hearing-in-noise performance (Otte et al., 1978; Makary et al., 2011). In cases of cochlear damage as a result of noise or drug exposure, some studies showed that while the loss of IHC occurred within a few days, the loss of dendrite and soma occurred within a few weeks and years, respectively (Liberman and Kiang, 1978; Hinojosa et al., 2001; Wu et al., 2019). A recent study reported up to 80% loss of connections between IHCs and ANFs for over 60-year-old human temporal bones, whereas only 10% of IHC degenerated (Wu et al., 2019). Many histological data of humans demonstrate that ANF degeneration mainly affects the dendrites (Nadol, 1990; Schuknecht, 1993). However, to our knowledge, no histological data are available on the dendritic degeneration in terms of diameter and myelination thickness based on the audiogram evaluations in humans. Our objective is to answer the question that how the degree and type of such ANF degeneration disturb the hearing efficiency with or without a CI.

This study investigated the ANF condition in terms of the three degeneration indicators: dendrite diameter (DD), myelination thickness (MT), and G-ratio (the ratio of the inner to the outer diameter of a myelinated fiber). Image data of four human cochleae at different ages with different hearing levels were used for a semi-automatic calculation of DD and MT of the same sample population in comparable regions in all specimens. Based on these data, a computer simulation was conducted for an electrical circuit model of an auditory neuron (Rattay et al., 2001). In the first simulation part, the model was used to mimic the synaptic excitation of the IHC. For a more realistic investigation, a noise channel was applied to the model to study the effect of dendritic degeneration on spike conduction. We showed that extremely thin fibers appeared in intense hearing loss cases, and they cause a reduction in spiking patterns and an increase in the delay of spike arrival times. In the second part of computer simulations, extracellular stimulation was evaluated for 30 three-dimensional ANF pathways from micro-CT recordings (Potrusil et al., 2020), assuming different levels of dendritic degeneration concerning DD and MT. The threshold profiles of the investigated ANFs for 10 electrode positions of a lateral versus a perimodiolar CI system were analyzed. We show that the spiking performance varies with the degeneration status of the neurons.

## Materials and Methods

### Preparation and Fixation

In this study, four individual human inner ears, one normal case (control) and three hearing loss cases (S1, S2, and S3) with hearing impairment differing in three cochlear regions based on the audiogram evaluations (Table 1), were used following ethical guidelines according to the Division of Clinical and Functional Anatomy of the Innsbruck Medical University (McHanwell et al., 2008; Riederer et al., 2012). Prefixation was done with the Karnovsky solution within 1–3 h after death by perilymphatic perfusion through the round and oval windows. During the autopsy, the temporal bones were removed and fixed by immersion and repeated perilymphatic perfusion. After post-fixation with 1.5% osmium tetroxide (OsO_4_) for 90 min and washing of the specimen, the excess bone around the cochlea was removed using a Bien Air CE 0120 driller until only a thin bony shell surrounded the cochlea. The temporal bones were dehydrated with 70% ethanol and embedded without removing the bony shell. Then the block-surface method (Spoendlin and Schrott, 1987) was used. A mid-modiolar cut was performed with a circular saw, and then small disks of the cochlear half-turns were dissected and divided into segments. The lamina was re-embedded, and tangential histological sections of the whole segment were evaluated by counting the cross-sectioned myelinated nerve fibers. The histological sections were done as near as possible to the organ of Corti. The number of myelinated nerve fibers was evaluated by counting the cross-sectioned fibers between osseous spiral lamina and organ of Corti of each segment.

**TABLE 1:** Specimen details on hearing deficits as provided by the Innsbruck Medical University, Department of Otorhinolaryngology, Laboratory for Inner Ear Biology.

Specimen ID	Audiogram basal (dB)	Hearing-loss level (basal)	Audiogram middle (dB)	Hearing-loss level (middle)	Audiogram apical (dB)	Hearing-loss level (apical)	Age (years)	Audiogram before death (months)
Control	17	Normal	7	Normal	11	Normal	47	10
S1	97	Profound	66	Severe	42	Moderate	67	5
S2	57	Moderate	28	Slight	17	Normal	63	15
S3	57	Moderate	20	Normal	20	Normal	80	1

### Evaluation of Hearing

Pure-tone audiograms were performed with a standard audiometer in a sound-attenuated room. Thresholds were measured between 0.125 and 8 kHz. The audiogram performed before death was used. The durations between the last audiogram and death were 1, 5, 10, and 15 months for S3, S1, control, and S2, respectively. Pure-tone averages were calculated for the low (0.125–0.5 kHz), mid (1–2 kHz), and high (4–8 kHz) frequencies. The degree of hearing loss was determined using the WHO criteria (25 dB or better, no impairment; 26–40 dB, slight impairment; 41–60 dB, moderate impairment; 61–80 dB, severe impairment; 81 dB or greater, profound impairment including deafness) (World Health Organization [WHO], 1991). The frequency ranges of 0.125–0.5, 1–2, and 4–8 kHz were assigned to the apical, middle, and basal regions of the cochlea, respectively.

### Imaging, Data Processing, and Statistics

Based on the block-surface method (Spoendlin and Schrott, 1987), it is feasible to assess the cochlea quantitatively at a light and electron microscopic level within the osseous spiral lamina and the cochlear nerve. ANFs of all specimens were divided into equal and comparable segmented regions, from basal to apical. Sections were digitized using a Zeiss Axio Imager.M2 equipped with a Zeiss Axiocam 512. Full-resolution images were acquired with a Plan Apochromat 63 × 1.4 lens and used to evaluate DD, MT, and G-ratio of the ANFs. Data processing was done with ImageJ as well as MATLAB (version R2018b)^1^, using a toolbox (Zaimi et al., 2016) for semi-automatic segmentation to investigate the dendrite of the ANFs. For a systematic comparison, an equal number of ANFs were counted in all three regions.

Statistical analyses were conducted using MATLAB and Python programming language (version 2.7)^2^. All data shown in Table 1 were collected based on the degree of hearing loss at three regions of the basal, middle, and apical with 450, 520, and 150 fiber numbers, respectively. The data were compared with the healthy specimen as control. The Kruskal–Wallis test was used to determine if there were significant differences between groups in each region, separately. Pairwise Conover test was applied to compare the medians of all groups.

### Computational Model and Simulations

**FIGURE 2:**
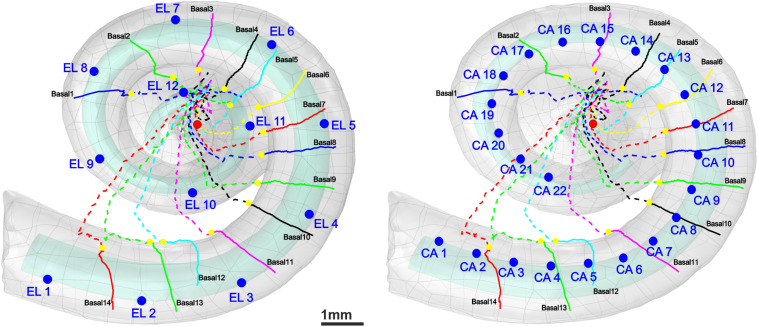
3D model of scala tympani including 12 electrodes of a lateral CI **(left)** and 22 electrodes of a perimodiolar CI (that is close to the center, **right**) as well as the 14 pathways of ANFs in the basal turn. The dendrites, soma positions, and axons of the nerve pathways are represented with colored solid lines, yellow spheres, and colored dashed lines, respectively. For the sake of clarity, all electrode positions are shown as blue spheres although in calculations each active electrode is a hemisphere. The axis of modiolus is marked as red sphere.

Auditory nerve excitation was simulated by Rattay’s multi-compartment model described in Rattay et al. (2001). More details about the geometry and kinetics of the model can be found in Potrusil et al. (2020). However, the geometrical parameters of the dendrites with internode lengths of 250 μm (except for the last internode) and 2.5-μm-long nodes of Ranvier were applied to the model to simulate the ANFs according to the 30 three-dimensional pathways and soma positions. Simulations were performed in two parts using MATLAB. In the first part, a 100-μs monophasic pulse was applied for intracellular stimulation to mimic “natural” ANF firing via synaptic excitation from an IHC. Our pulse is essentially shorter than the 280-μs rise time of postsynaptic currents observed in rat experiments but with peak values of 400 pA (Grant et al., 2010); such recorded currents at the IHC ribbon synapse are about 15 times stronger than the threshold current (Rattay et al., 2013), and they generate the AP within ∼100 μs (Rattay and Danner, 2014). To simulate the latency variations, a noise channel was added, quantified by the parameter *k*_*noise*_ = 0.05 μA mS^–1/2^ (Rattay et al., 2001). This approach assumes that every compartment’s noisy current component is related to the number of sodium ion channels in the cell membrane. A biphasic (cathodic first) and a monophasic (cathodic) pulse of 100 μs per phase were applied to investigate the threshold profile for extracellular stimulation in a cochlear implant environment. In this case, an anatomically detailed finite element model of a human cochlea along with 30 reconstructed tonotopically organized auditory pathways of type I auditory nerve bundles from our previous work (Potrusil et al., 2020) was implemented in COMSOL Multiphysics (version 5.5)^3^. In addition, two different electrode arrays, a lateral and a perimodiolar CI system, were modeled and added to our cochlea model to evaluate the excitation profiles of the ANFs. Both CI models are designed according to manufacturer data of a deep-insertion array of FLEX SOFT, MED-EL, Innsbruck, Austria (12 electrodes; most basal EL1, most apical EL12) and a perimodiolar located array of CI24RE Contour Advance, Cochlear, Sydney, Australia (24 electrodes; most basal CA1, most apical CA22). Figure 2 shows the top views of both electrode arrays inserted into the specimen’s scala tympani. For better understanding, the basal nerve pathways and electrode positions are added to the figure. Table 2 represents the measured angles from the round window with respect to the modiolus axis for 10 investigated electrodes (five from each CI array system), as well as the fiber with the closest distance from its dendritic terminal to the center of the electrode named target neuron (TN).

**TABLE 2:** Angles of the investigated electrodes and the corresponding target neurons (TN) measured from the round window with respect to modiolus axis.

Lateral electrode array	Electrode angle (°)	Perimodiolar electrode array	Electrode angle (°)	TN	TN angle (°)
EL1	10	CA2	15	Basal14	28
EL3	69	CA6	73	Basal11	79
EL5	143	CA11	153	Basal7	137
EL8	295	CA19	317	Basal1	298
EL9	343	CA20	332	Middle9	327

## Results

### Variations in Dendrite Diameter and Myelination Thickness

**FIGURE 3:**
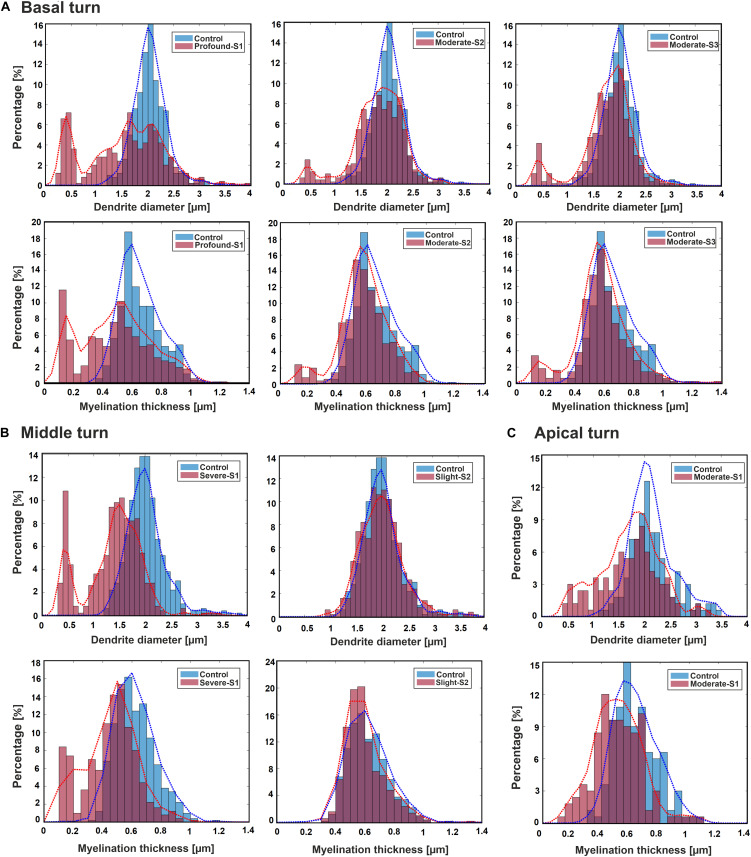
Histograms and fit kernel distributions (dashed lines) for DD and MT of the ANFs for control and one specimen with determined hearing loss level, calculated in the basal turn **(A)**, middle turn **(B)**, and apical turn **(C)**.

Figure 3 displays histograms for DD and MT for each hearing loss case compared with the control in the three cochlear regions basal, middle, and apical. In addition, the fit kernel distribution was used for better comprehension. The unimodal control data spread from about 1.3 and 0.35 μm to 3.5 and 1.2 μm with peaks occurring at about 2 and 0.6 μm for DD and MT, respectively, in all three regions. The MT values are clustered on the right side of the histogram, demonstrating a right-skewed form.

In the basal turn (Figure 3A), in S1 (Profound impairment), DD spreads from 0.3 to 3.5 μm with peaks at about 0.4, 1, 1.5, and 2 μm showing a multimodal distribution whereas MT spreads from about 0.1 to 1.1 μm with peaks occurring at about 0.1, 0.3, and 0.55 μm. Both parameters are wider, and more spread out in the S1 case, indicating more variety. S2 and S3 (Moderate impairment) represent similar trends with spreading from about 0.4 and 0.15 μm to 3.5 and 1.1 μm for DD and MT, respectively. DD distributions display a multimodal form with peaks at about 0.5, 1.5, and 2 μm, whereas MT represents a bimodal behavior with peaks at about 0.2 and 0.55 μm for both cases.

In the middle turn (Figure 3B), in S1 (Severe impairment), DD expands from 0.3 to 3.5 μm with peaks at about 0.4, 1.3, and 2 μm representing a multimodal distribution behavior, whereas MT spreads from about 0.1 to 1.1 μm with similar peaks as the profound case of the basal turn. In S2 (Slight impairment) case, DD expands from about 1 to 3.5 μm, and MT has the same distribution as the control.

Figure 3C demonstrates the apical turn; S1 (Moderate impairment) shows the data spreading from about 0.4 and 0.15 μm to 3.5 and 1.1 μm in DD and MT, respectively. The peaks arise in about 0.5, 1.5, and 2 μm in DD, and 0.5 μm in MT.

**FIGURE 4:**
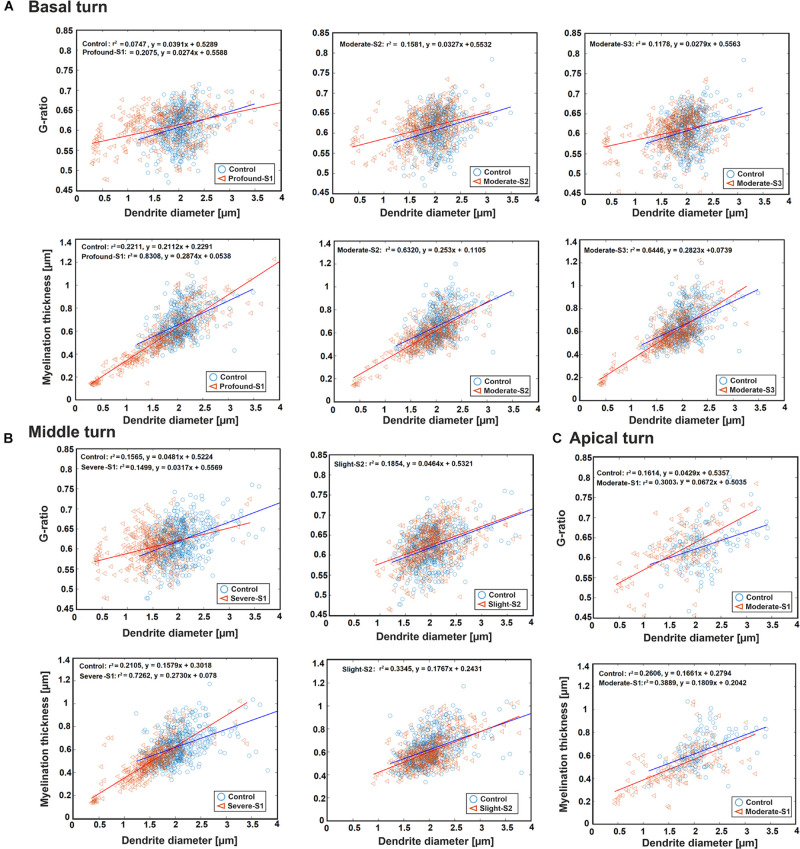
Scatter plot for the G-ratio and MT against DD of the ANFs for control and one specimen with determined hearing loss level, calculated in the basal turn **(A)**, middle turn **(B)**, and apical turn **(C)**. The best-fit linear regression to each scatterplot is included.

Figure 4 represents the scatter plot with the best-fit linear regression of the G-ratio and MT against DD in all three regions between the control and a hearing loss case. In the basal turn, the G-ratio shows a weak linear relationship with DD in all cases, whereas MT and DD have a strong linear relationship with *r*^2^ = 0.83, 0.63, and 0.64 for S1 (Profound impairment), S2, and S3 (Moderate impairment), respectively, and a weak relationship (*r*^2^ = 0.22) for the control (Figure 4A). In addition, there was a significant difference in DD and MT (*p* < 0.001) in all three hearing loss cases versus the control. However, the same significant difference in G-ratio was found only between the control and the profound case.

In the middle turn, the G-ratios in all three cases, as well as the MT in control and the slight case, show a weakly positive linear relationship with DD. On the other hand, MT and DD have a strongly positive linear relationship (*r*^2^ = 0.73) in the severe case (Figure 4B). Moreover, the significant difference in all three parameters (*p* < 0.001) was found between the control and the severe case.

In the apical turn, a weakly positive linear relationship was found for G-ratio and MT with DD in both cases (Figure 4C). In addition, a significant difference (*p* < 0.001) was only observed in DD and MT between the control and the moderate case.

### Impact of DD and MT on Synaptic Threshold Current and AP Arrival Time in the Axon

**FIGURE 5:**
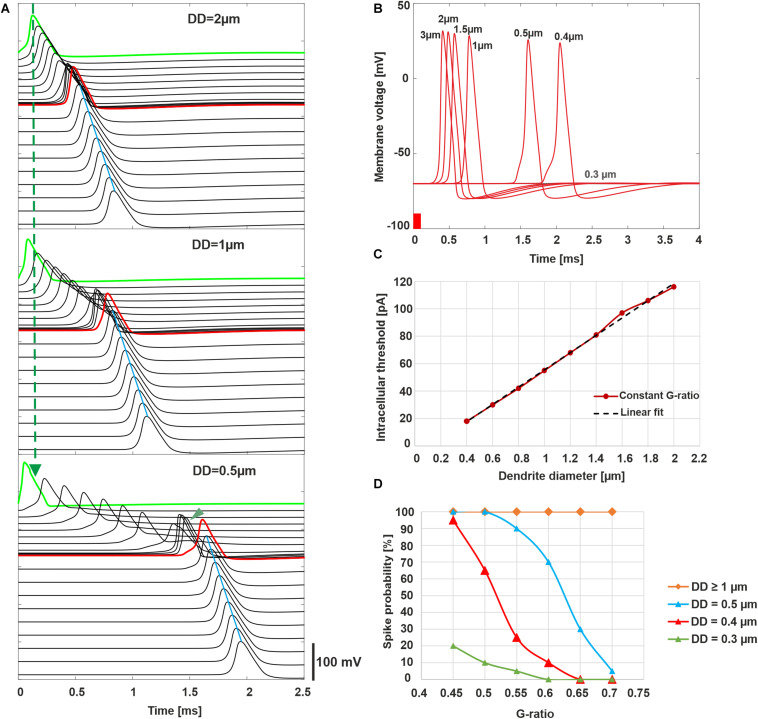
Intracellular ANF stimulation simulating synaptic excitation. **(A)** Excitation and AP conduction along the ANF (300 pA, 100 μs). AP is initiated in the first compartment (green; note the quicker excitation when DD is reduced marked by dashed vertical line; presomatic compartments are marked with arrowhead for DD = 0.5 μm and show slow conduction velocity for thin fibers). Conducted APs are shown for all active compartments with a vertical shift indicating the compartment’s location. The arrival time of the AP in the soma (red) increases when DD decreases. Axon’s conduction velocity (marked by blue line at AP peaks) outmatches all dendritic cases as a consequence of the larger axon diameter (4 μm). **(B)** Membrane voltage in the soma for varied DD demonstrates increased latency when DD is reduced; no AP for DD = 0.3 μm. **(C)** Synaptic threshold current for AP conduction as a function of DD (red) and linear fit (black dashed line). **(D)** Probability of spike conduction across the soma as a function of G-ratio for various DD. In **panels A–C**, G-ratio = 0.625 (mean value of the control). **(A)** and **(B)** were calculated without noise, **(C,D)** with the stochastic model that included current fluctuations in the active membrane.

Our first computer experiments concentrate on the impact of DD and MT on the excitation and the arrival time of the AP at the soma for physiological hearing in contrast to stimulation with a cochlear implant. Synaptic IHC excitation and spike conduction along the ANF were simulated by a 100-μs pulse of 300 pA injected into the first compartment (Figure 5A). Thin fibers need weak postsynaptic currents to generate a spike, and consequently, a fixed stimulus amplitude causes earlier AP peaks for smaller DD. Comparing green APs depicting first compartment excitations, the vertical dashed line demonstrates earliest AP peak for the thinnest diameter in Figure 5A. In contrast to this advantage for thin fibers, the AP conduction velocity of myelinated fibers is proportional to fiber diameter. Tiny fibers lose any temporal benefit of AP generation during slow AP conduction (nine black lines for six nodes of Ranvier and three non-myelinated presomatic compartments) before AP arrives at soma (red line, Figure 5A). The impact of DD on the AP conductance time to the soma is rather small for dendrites of the control group in contrast to the first part of all presented multimodal histograms (Figures 3, 5B). The threshold current for AP initiation is inverse to DD (Figure 5C) because small myelinated fibers need small intra-cellular current flow for AP conductance (Rattay, 1990). However, the high capacitance of the soma cannot be loaded with such a small intracellular current delivered by DD < 0.4 μm, although there is support from a non-myelinated presomatic region. This presomatic region, represented by three compartments and marked by the arrowhead in the lowest graph of Figure 5A, amplifies the intracellular current flow but causes an additional AP delay at soma that increases when DD is reduced (compare the middle and lower graph of Figure 5A). In conclusion, as the fiber diameter decreases, the AP needs more time to pass the soma, but in our deterministic model, AP conduction into the axon is not feasible for fibers with DD less than 0.4 μm.

To estimate the probability of spike conductance as functions of DD and G-ratio, ion current fluctuations were included in all active membrane compartments, and 20 runs were performed with an amplitude of 300 pA for all cases. Although the probability of spike arrival in the axon is 100% for DD larger than 1 μm, it reduces by decreasing DD (Figure 5D).

**FIGURE 6:**
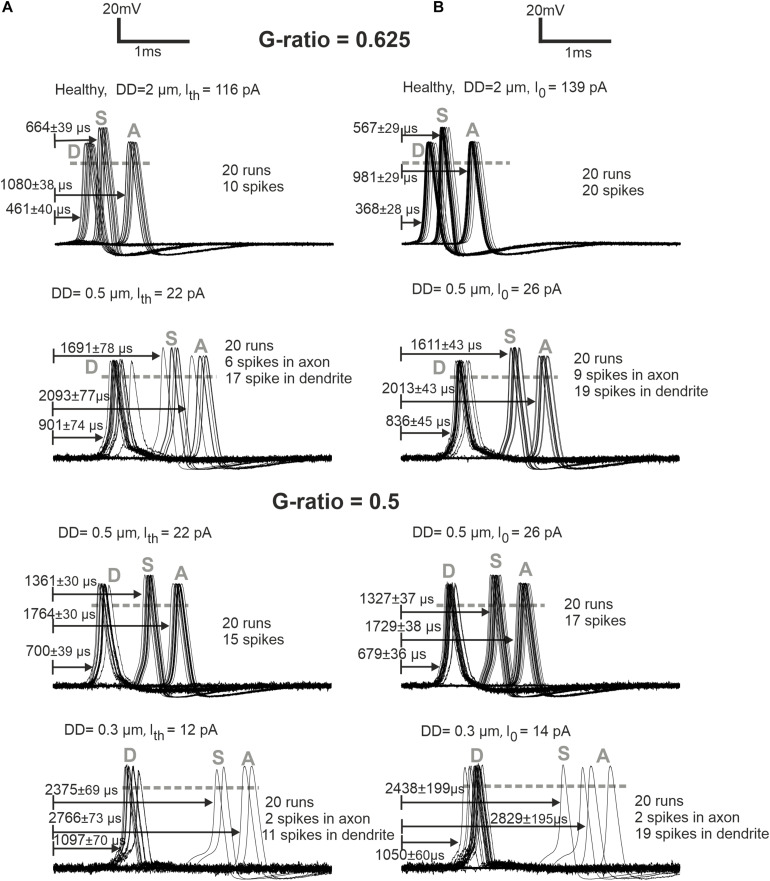
ANF responses at dendrite (first compartment), soma, and axon (last compartment) are marked with D, S, and A, respectively. **(A)** Later APs and larger jitters for synaptic stimuli at threshold (*I*_*th*_) versus **(B)** more synchronized responses for synaptic stimuli at *I*_0_ = 1.2 × threshold are represented for G-ratios = 0.625 and 0.5. While the arrows start at the stimuli onset, the delays represent the time when the membrane voltage reaches 0 mV (shown with gray dashed horizontal lines). Recording point A of simulations corresponds with the axonal terminal from the micro-CT sample (Potrusil et al., 2020).

Loss of APs was also investigated for weak stimulation, namely, at the threshold and 1.2 times threshold (Figure 6). The normal (standard) fiber (2 μm) shows a sharply synchronized spiking pattern with a reduced delay for the higher amplitude. Increasing the amplitude causes a decrease in jitter and delay. The case of DD = 0.5 μm demonstrates that synchronization becomes higher, and the delay becomes shorter when the G-ratio is reduced (increasing of MT). For the higher G-ratio, only 30–45% of spikes can pass the soma. The case DD = 0.3 μm generates no AP for G-ratio higher than 0.55 (simulated but not shown). However, by shortening the internodal lengths to half of its standard length (250 μm), the AP is generated in the dendrite even with the standard G-ratio of 0.625, yet the small intracellular current is unable to load the soma capacitance enough to initiate a spike in the soma.

**FIGURE 7:**
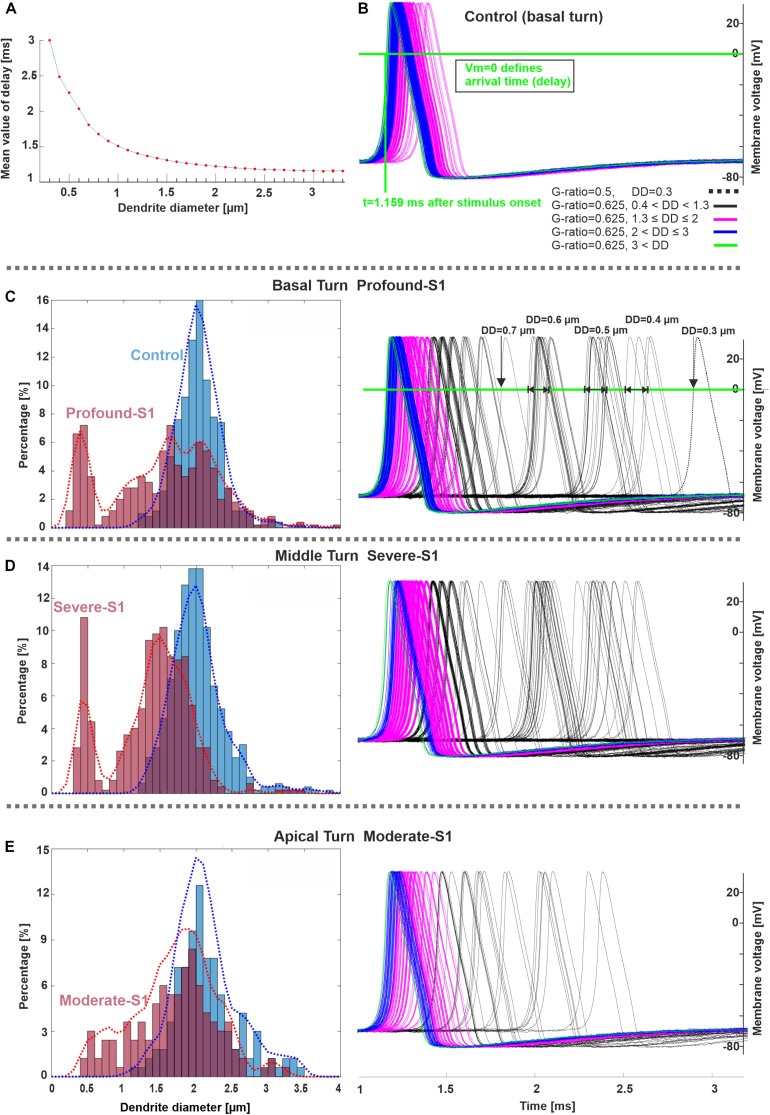
Spike pattern comparison between control and hearing loss cases in three cochlear regions. **(A)** Delay (mean values) as a function of DD (20 runs per DD). **(B–E)** Spiking patterns of the investigated specimens (S1 vs. control) based on the number of counted fibers in different cochlear regions, basal, middle, and apical stimulated with 300 pA. The horizontal and vertical green lines in **panel B** represent the membrane voltage value at 0 mV and the corresponding time (delay) at soma after the stimulus onset, respectively. The color code in panel **(B)** applies to **(C–E)**. Recording point at axonal terminal. The *x*-axis of the right panel in **panel E** applies to all spike pattern graphs.

The next experiments show the expected AP delays in the soma as a function of DD (Figure 7A) and according to the DD histograms of Figure 3 (Figures 7B–E). The largest delay and jitter values occur for very thin fibers found in extreme hearing loss cases (Table 3). For the thickest group of fibers (DD > 3 μm), the mean delay and jitter values are smallest compared with the other fiber groups. The successful spike conduction of thicker fibers is based on a high density of sodium channels in the nodes of Ranvier and an adequate number of insolating myelination sheets covering the internodes which limit the transmembrane currents.

**TABLE 3:** Mean delay ± STD (jitter) for the counted fibers in each specimen.

DD (μm)	Mean delay ± STD (ms)			
	Control (basal)	Profound (basal)	Severe (middle)	Moderate (apical)
0.3 ≤ DD < 1.3	–	1.816 ± 0.405	1.800 ± 0.387	1.664 ± 0.257
1.3 ≤ DD ≤ 2	1.248 ± 0.032	1.279 ± 0.044	1.284 ± 0.043	1.270 ± 0.045
2 < DD ≤ 3	1.197 ± 0.014	1.191 ± 0.016	1.201 ± 0.013	1.192 ± 0.015
DD > 3	1.159 ± 0.006	1.162 ± 0.010	1.163 ± 0.007	1.157 ± 0.006

*For the calculation, an intracellular stimulus of 300 pA and various run numbers, based on each fiber group quantity, are chosen.*

### Extracellular Stimulation

**FIGURE 8:**
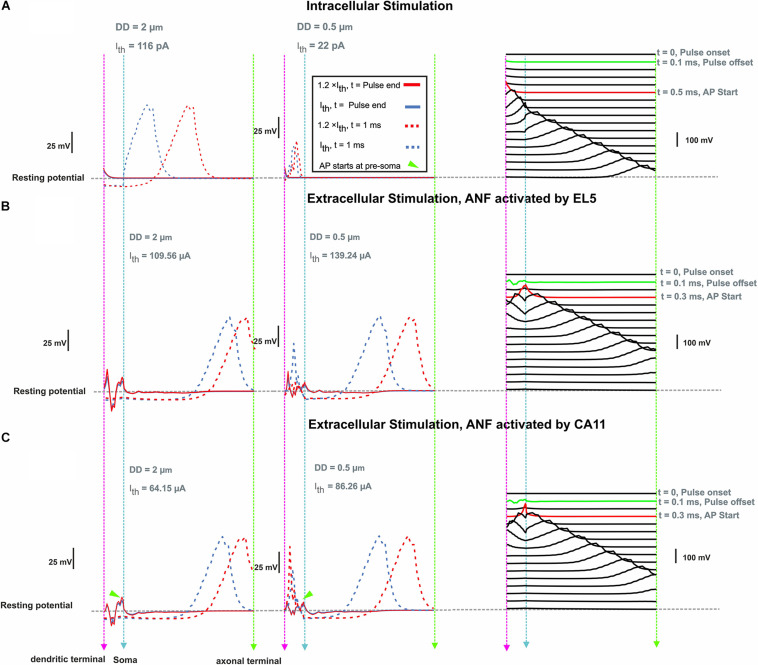
Membrane voltage along ANF basal7 (143°, G-ratio = 0.625) as a response of intra- and extracellular stimulation. **(A)** Membrane voltage for DD = 2 vs. 0.5 μm at pulse offset (0.1 ms) and 1 ms after intracellular stimulus onset with a pulse amplitude at threshold (*I*_*th*_) and 1.2 × threshold. Right panel: AP propagation along the ANF with DD = 2 μm at threshold. Each line shows the membrane voltage at a fixed time. The green and red lines represent the times corresponding to stimulus offset and the first achievement of the AP peak, respectively. **(B)** Same configuration for extracellular stimulation of lateral electrode EL5 and **(C)** perimodiolar electrode CA11. Vertical dashed pink, cyan, and green lines represent the positions of the dendritic terminal, soma, and axonal terminal, respectively. Arrowheads indicate the firing start at presoma.

For ANF basal7 (in the position 137°), synaptic excitation of a monophasic anodic pulse and extracellular stimulation of a monophasic cathodic pulse from EL5 (143°) and its corresponding perimodiolar electrode CA11 (153°) were compared (Figure 8). In contrast to synaptic excitation, where the AP is initiated in the first compartment, it is more complicated to understand the AP generation and propagation during extracellular stimulation. Depending on the cell geometry, cochlear region, electrode position, and pulse intensity, there are several sites along the fiber where spikes can be generated and, moreover, excitation is possible both with cathodic (negative) and anodic (positive) pulses. An indicator for possible spike initiation sites is the membrane voltage profile along the ANF under subthreshold conditions, e.g., the full blue line in Figures 8B,C, which has its maximum within different parts of dendrite for both DD = 2 μm (left) and DD = 0.5 μm (middle). As mentioned before, thinner fibers are easier to excite with intracellular stimuli, but the opposite, known as reversed recruitment order (Blair and Erlanger, 1933), is seen for extracellular stimulation (compare values of threshold currents in Figures 7A–C).

**FIGURE 9:**
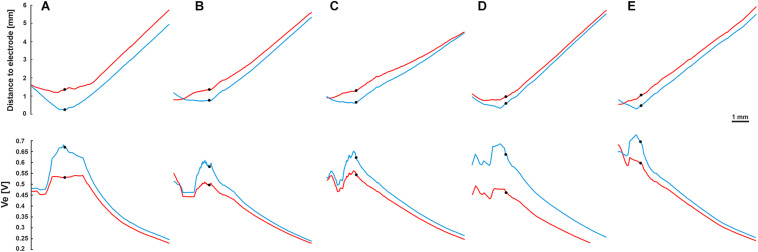
Fiber distance to the stimulating electrode (upper panel) and extracellular voltage *V*_*e*_ (lower panel) versus length of the fibers. Blue and red curves for the perimodiolar and lateral electrodes, respectively. **(A)** EL1 and CA2, TN: basal14; **(B)** EL3 and CA6, TN: basal11; **(C)** EL5 and CA11, TN: basal7; **(D)** EL8 and CA19, TN: basal1; and **(E)** EL9 and CA20, TN: middle9. *V*_*e*_ is inversely related to the distance to the electrode. Black circles indicate soma positions, 1 V was applied to the surface of the active electrode.

To investigate the impact of the neural pathway on the threshold profiles, five electrodes from each CI array system were selected (Table 2 and Figure 2). A fixed potential of 1 V was applied at the surface of the active electrode, and the extracellular voltage (*V*_*e*_) distribution along the interested pathways was calculated with our finite element model. Figure 9 shows the electrode distances (upper panel) and the inverse relationship of *V*_*e*_ (lower panel) along the fiber of five TNs in blue and red, which were stimulated with perimodiolar and lateral CI systems, respectively.

**FIGURE 10:**
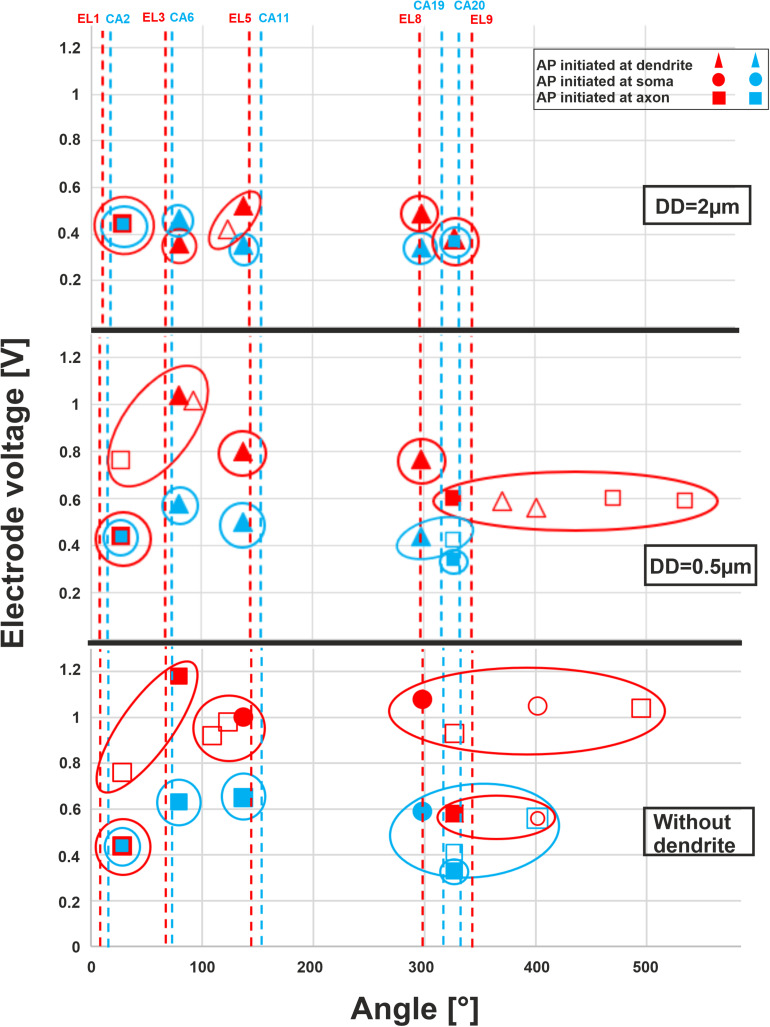
Excitation thresholds of TNs and co-stimulated ANFs along the fiber angles calculated for 10 electrodes. The electrode thresholds for three DD of 2, 0.5, and 0 μm (without dendrite) is demonstrated in the upper, middle, and lower panel, respectively. Note that all investigated ANFs are considered with the same dendrite, soma, and axon diameters at each level of calculation. Vertical dashed lines indicate the angle of electrodes, filled symbols TN, unfilled co-stimulated neurons (blue for perimodiolar, red for lateral CI system).

The threshold profiles for the selected electrodes are shown in Figure 10 for biphasic (cathodic first) stimulation with 100 μs per phase. Applying the threshold current of the closest TN (ANF 28°), the basal electrodes (EL1 and CA2) stimulated only one of the 30 investigated fibers. There was no co-stimulated ANF in this region. The threshold is the same for the three investigated DD (2, 0.5 μm, and without dendrite) because the AP is initiated in this case at the axon. Contrarily, the second investigated basal electrode from the lateral array (EL3) stimulated another group of fibers before the TN basal11 (79°) in the case of DD = 0.5 μm and without dendrite, whereas the corresponding perimodiolar electrode (CA6) stimulates only the TN.

For the middle electrodes EL5 and CA11, co-stimulation of ANFs depends on dendrite condition and CI system: EL5 stimulated another group of fibers before TN basal7 (137°) in the case of DD = 2 μm, but no co-stimulation when DD = 0.5 μm and two co-stimulated neurons under the assumption that ANFs are without dendrites. For each level of degeneration, we assumed that all 30 ANFs had the same dendrite, soma, and axon diameter and, subsequently, different co-stimulated neurons might appear when only the DD parameter is changed. For instance, basal6 is a co-stimulated neuron in the case of lateral electrode EL5 when DD is 2 μm. However, this ANF does not appear in the next level of degeneration (DD = 0.5 μm) because its electrode voltage threshold is essentially larger than the threshold of TN, basal7 (Figure 10, upper and middle panels).

In the upper-middle turn, we compared active electrodes EL8, EL9, with CA19, CA20, respectively. The stimulation of ANFs of this region becomes less selective mostly for the lateral electrode system when dendrite degeneration increases (Figure 10, middle and lower panels). With increasing dendrite degeneration level, higher thresholds are needed to activate the TN, leading to disruption of the electrode selectivity and cochlea tonotopic function in this region.

Table 4 shows threshold values, spike initiation sites, and co-stimulated ANFs. The values increase with increasing degeneration level of the TN, resulting in stimulating more neurons in the neighborhood, particularly in the case of the lateral electrode system. The mean values of standard deviation (STD) for threshold variations of the investigated electrodes during dendritic degeneration of each TN were 0.22 V for lateral electrode array and 0.07 V for perimodiolar array. Moreover, the perimodiolar array requires a lower threshold than the lateral electrode system in most cases, which is in line with findings in clinical studies (Cohen et al., 2001; Long et al., 2014; Lee et al., 2019).

**TABLE 4:** Thresholds of TNs and the co-stimulated neurons for 10 investigated electrodes for the lateral and perimodiolar electrode array systems.

Electrode array	TN	Threshold (V)	AP location TN	Co-stimulated neuron	Threshold (V)	AP location co-stimulated
**DD = 2 μm**						
EL1	Basal14	0.44	Central	–	–	–
EL3	Basal11	0.36	Dendrite	–	–	–
EL5	Basal7	0.52	Dendrite	Basal8	0.42	–
EL8	Basal1	0.49	Dendrite	–	–	–
EL9	Middle9	0.38	Dendrite	–	–	–
**DD = 0.5 μm**						
EL1	Basal14	0.44	Central	–	–	–
EL3	Basal11	1.04	Dendrite	Basal14/basal10	0.76/1.02	Central/central
EL5	Basal7	0.8	Dendrite	–	–	–
EL8	Basal1	0.77	Presoma	–	–	–
EL9	Middle9	0.6	Central	Middle7/middle3/middle8/middle5	0.56/0.59/0.59/0.6	Presoma/central/presoma/postsoma
**Without dendrite**						
EL1	Basal14	0.44	Central	–	–	–
EL3	Basal11	1.18	Central	Basal14	0.76	Central
EL5	Basal7	1	Soma	Basal9/basal8	0.92/0.98	Central/central
EL8	Basal1	1.08	Soma	Middle9/middle4/middle7	0.93/1.04/1.05	Central/central/soma
EL9	Middle9	0.58	Central	Middle7	0.56	Soma
**DD = 2 μm**						
CA2	Basal14	0.44	Central	–	–	–
CA6	Basal11	0.46	Dendrite	–	–	–
CA11	Basal7	0.35	Presoma	–	–	–
CA19	Basal1	0.34	Dendrite	–	–	–
CA20	Middle9	0.37	Central	–	–	–
**DD = 0.5 μm**						
CA2	Basal14	0.44	Central	–	–	–
CA6	Basal11	0.58	Presoma	–	–	–
CA11	Basal7	0.5	Presoma	–	–	–
CA19	Basal1	0.44	Dendrite	Middle9	0.42	Central
CA20	Middle9	0.34	Central	–	–	–
**Without dendrite**						
CA2	Basal14	0.44	Central	–	–	–
CA6	Basal11	0.63	Central	–	–	–
CA11	Basal7	0.65	Central	–	–	–
CA19	Basal1	0.59	Soma	Middle9/middle7	0.41/0.56	Central/central
CA20	Middle9	0.33	Central	–	–	–

## Discussion

Only a small number of human subject studies were performed on the morphometry of degenerated ANFs. Analysis of the signaling of the human ANFs is of great interest to pathophysiology and a better understanding of CI stimulation. In the first part of this study, four human cochleae with normal hearing and different hearing loss levels were investigated, and their ANFs were evaluated concerning diameters and myelin thickness of their dendrites. The extracted morphometric data were used as input in a multi-compartment model to compare neural signal conduction in normal and degenerated TNs for synaptic excitation from IHCs. In addition, two CI systems were added to our finite element model to investigate the impact of dendrite degeneration on signaling during extracellular stimulation.

### Variation in Dendrite Diameter

Unimodal distribution in dendritic ANF diameters of mammalian species such as cat, guinea pig, and monkey was reported (Gacek and Rasmussen, 1961; Arnesen and Osen, 1978; Friede, 1984; Gleich and Wilson, 1993). Similar to animals, Spoendlin and Schrott reported unimodal diameter distribution for humans with normal hearing (Spoendlin and Schrott, 1989). The group has reported the same range of diameter for three cochlear regions, basal, middle, and apical. They suggested this similarity results from the almost similar dendritic length of human ANFs in all three regions compared with other species. The current study shows a unimodal distribution in normal hearing specimen versus multimodal distribution for hearing loss cases (Figure 3). Our reported diameter range for the normal hearing case is in agreement with our recent study in all three cochlear regions (Potrusil et al., 2020).

### Appearance of Small Fibers in Hearing Loss Specimens and Consequences

A massive number of fibers with DD < 1 μm was found in subjects with high levels of hearing loss and poor speech discriminations (Spoendlin and Schrott, 1989). We found similar fiber ranges in our hearing loss cases and extremely thin fibers (smaller than 0.5 μm) in severe and profound cases. Dendrite diameter variation leads to changed spike conduction velocity that may affect speech discrimination (Rattay and Motz, 1987; Rattay et al., 2013). According to our modeling study, the spike conductance of such thin dendrites with G-ratio = 0.6 (about mean value of G-ratio) resulted in loss of signals and thus in a significantly reduced axonal spiking probability, e.g., 10% (DD = 0.4 μm, Figure 5D); jitter and spike latencies increase versus control up to 0.4 and 1 ms, respectively (Figure 7 and Table 3). Such high delays in spike patterns (see Figures 7B vs. C) are expected to affect the temporal fine structure in the neural pattern of the cochlear nerve and, consequently, auditory perception (Rattay and Lutter, 1997). These extended latencies and jitters, calculated for intracellular stimulation to mimic the synaptic excitation, affect also the neural status between the ears and by larger variations in interaural time differences they contribute to poor sound localization and speech discrimination, especially in a noisy environment (Zwislocki and Feldman, 1956; Rosen, 1992; Saberi and Perrott, 1999; Bernstein and Trahiotis, 2015).

### Degeneration Level Affects Cochlear Implant Outcomes

Techniques such as electrocochleography, polarity sensitivity, electrically evoked compound action potential (eCAP), and eCAP responses to changing interphase gap are used to evaluate the residual neural health (Santarelli et al., 2008; Garadat et al., 2012; Long et al., 2014; Pfingst et al., 2015; Hughes et al., 2018; Shearer et al., 2018; He et al., 2020). A recent study on lateral and perimodiolar CI systems reported a lower eCAP threshold in the apical region (Lee et al., 2019). Although their findings are in agreement with some studies (Cohen et al., 2001; Parkinson et al., 2002), others found the opposite result for the apical region (Polak et al., 2004; Brill et al., 2009; Van de Heyning et al., 2016). Table 4 demonstrates that both higher and lower thresholds in the apical turn are possible, but the correlation depends on the cochlear geometry, electrode position, neural status, and degeneration level of the ANFs.

In a study of 10 cats, better localized neural excitation for electrodes close to the modiolus compared with the lateral wall was reported (Shepherd et al., 1993). Similar investigations were performed for human subjects that suggest lower threshold, better localization of the nerve stimulations, broader dynamic range, and less channel interactions are possible for electrode array systems close to modiolus compared with those close to the lateral wall of scala tympani (Cohen et al., 2001; Donaldson et al., 2001; Tykocinski et al., 2001; Saunders et al., 2002; Hughes and Abbas, 2006; Lee et al., 2019). In agreement, we showed lower thresholds for the TNs in most investigated electrodes located close to modiolus compared with the lateral array (Table 4). According to our findings, insignificant channel interaction (concerning the tonotopic principle) occurred for lateral CI and no interaction for perimodiolar array in case of fibers with DD = 2 μm (Figure 10, upper panel), whereas with severe levels of degenerations, a lateral CI shows poorer focal stimulation efficiency compared with a perimodiolar CI (Figure 10, middle and lower panels).

### Clinical Implications

In studies related to cochlear insults such as noise and drug exposure, it was reported that soma and axonal degeneration were less and slower than the loss of IHCs and dendrites (Johnsson, 1974; Johnsson and Hawkins, 1976; Hinojosa et al., 2001). On the other hand, in a series of histological studies by Spoendlin and Schrott (1988; 1989; 1990), they found that the number of dendrites highly correlated with the number of somata in hearing loss as well as normal-hearing specimens. These findings suggest that simply excluding the dendrite is no general rule, and the type of degeneration is highly dependent on the origin of the impairment. However, recent valuable models in computational studies mostly eliminate the dendrite to simulate the degeneration of ANFs (Briaire and Frijns, 2006; Snel-Bongers et al., 2013; Kalkman et al., 2014). Our study may shed more light on the impact of ANF degeneration on neural signaling, demonstrating that more realistic models should be considered for improving CI performance.

### Modeling Details and Limitations

The goal of our study was to find trends in the excitability of human ANFs when their dendrites are degenerated. Limitations of this study were a small number of available human cochleae, missing geometric parameters such as internode lengths, positions, and surfaces of nodes of Ranvier, lengths of pre-somatic sections, as well as complete individual data sets for every ANF concerning the connected soma and axon. Due to lack of these data, for all ANFs, we simply assumed constant values for the length of the non-myelinated presomatic section (100 μm), and the diameters of the spherical soma (20 μm) and the axon (4 μm) independent of the degree of degeneration. However, in a previous study, a quite constant diameter ratio of 2 was found for healthy subjects for (axon diameter)/(dendrite diameter) in the whole cochlea (Rattay et al., 2013). The vagueness on these post-dendritic data affects predictions such as “Is a spike elicited in the dendrite, soma, or axon?”

Other points of interest were the choice of the membrane model and the estimate of spiking probability. Biophysically based neuronal membrane models typically follow the pioneers Hodgkin and Huxley to quantify the ionic transmembrane currents via gating probabilities (Rattay et al., 2003). This method uses deterministic probabilities, which means running a model several times under the same conditions always produces exactly the same results. As spikes simulated with this method will not create any jitter seen in experiments, we added a noise term which can be interpreted as current fluctuations in the ANF (Hochmair-Desoyer et al., 1984; Rattay, 2000). Although other models for human ANF membranes are published, a review (Bachmaier et al., 2019) and some tests showed that our modified Hodgkin–Huxley model replicates many features known from experiments (Motz and Rattay, 1986; Rattay et al., 2013; Rattay and Danner, 2014). However, features not included in this simple membrane model are, e.g., accommodation effects during repetitive stimulation (Boulet et al., 2016) or the impact of *I*_*h*_ currents, which influence excitation during hyperpolarization phases (Hossain et al., 2005; Negm and Bruce, 2014).

Despite the mentioned shortcomings, combining the finite element approach with our compartment model is in good quantitative agreement with experimental CI data. Our calculated threshold current range for the active electrode was from 40 to 300 μA depending on the electrode array system and level of degeneration. This range is in agreement with clinical data from Kawano et al. (1998); van den Honert and Kelsall (2007), and Long et al. (2014). In addition, the intracochlear potential decrement along a CI array of our standard conductivity parameters was in line with clinical data from Tang et al. (2011) and Kalkman et al. (2014).

## Data Availability Statement

The original contributions presented in the study are included in the article/supplementary material, further inquiries can be directed to the corresponding author.

## Ethics Statement

The studies involving human participants were reviewed and approved by the Division of Clinical and Functional Anatomy of the Innsbruck Medical University. Written informed consent for participation was not required for this study in accordance with the national legislation and the institutional requirements.

## Author Contributions

AH and SS contributed to the conception, design of the study, design of FE model and computational model, data analysis, visualization, and manuscript writing and editing. LJC contributed to imaging data collection and clinical preparation. NF contributed to image acquisition, imaging data collection, and clinical preparation. AS-F contributed to design clinical experiments, clinical preparation, funding acquisition, and supervision. FR contributed to the conception, design of the study, data analysis, article writing and revising, as well as supervision. All authors contributed to the article and approved the submitted version.

## Conflict of Interest

The authors declare that the research was conducted in the absence of any commercial or financial relationships that could be construed as a potential conflict of interest.

## Footnotes

1 https://mathworks.com

2 https://www.python.org

3 https://www.comsol.com

## Abbreviations

ANF: auditory nerve fiber
AP: action potential
CI: cochlear implant
DD: dendrite diameter
eCAP: evoked compound action potential
IHC: inner hair cell
MT: myelination thickness
STD: standard deviation
V_*e*_: extracellular potential.
